# A Multivariate Approach to a Meta-Analytic Review of the Effectiveness of the D.A.R.E. Program

**DOI:** 10.3390/ijerph6010267

**Published:** 2009-01-13

**Authors:** Wei Pan, Haiyan Bai

**Affiliations:** 1 Division of Educational Studies and Leadership, University of Cincinnati, P.O. Box 210049, Cincinnati, Ohio 45221, U.S.A; 2 Department of Educational Research, Technology, and Leadership, University of Central Florida, P.O. Box 161250, Orlando, Florida 32816, U.S.A.; E-Mail: hbai@mail.ucf.edu

**Keywords:** D.A.R.E., drug use, tobacco, alcohol, meta-analysis, research synthesis

## Abstract

The Drug Abuse Resistance Education (D.A.R.E.) program is a widespread but controversial school-based drug prevention program in the United States as well as in many other countries. The present multivariate meta-analysis reviewed 20 studies that assessed the effectiveness of the D.A.R.E. program in the United States. The results showed that the effects of the D.A.R.E. program on drug use did not vary across the studies with a less than small overall effect while the effects on psychosocial behavior varied with still a less than small overall effect. In addition, the characteristics of the studies significantly explained the variation of the heterogeneous effects on psychosocial behavior, which provides empirical evidence for improving the school-based drug prevention program.

## 1. Introduction

Drug abuse is a prevalent problem affecting young generations worldwide [1]. In response to this issue, many drug prevention programs have been implemented in schools. The Drug Abuse Resistance Education (D.A.R.E.) program is the largest, school-based drug prevention program in the United States and other countries as well [2]. The D.A.R.E. program originated in 1983 from a local drug prevention program jointly sponsored by a school district in Los Angeles and the city police department. By 2007, more than 36 million school children around the world, including 26 million children in the United States, participated in the school-based drug prevention program [2]. D.A.R.E. America [2] also reported that, in the past three years, about 1,000 communities started D.A.R.E. programs in their schools; and, as a result, more than 75 percent of American school districts and 43 countries around the world now incorporate a D.A.R.E. program. The increasing number of school districts adopting the D.A.R.E. program speaks to its long-lasting reputation, and it became so popular and significant that one day each year has been declared as the National D.A.R.E. Day by the United States Presidential Proclamation since 1988.

The D.A.R.E. program was designed to help elementary and junior high school students resist the peer-pressure of experimenting with drugs, tobacco, and alcohol. The D.A.R.E. program aims to reduce drug abuse among children by providing them with information that encourages them to make healthy decisions. Its effectiveness has been assessed by its two major outcomes: (a) the reduction of *drug use*, which includes tobacco, alcohol, marijuana, and other illicit drugs; and (b) the improvement of *psychosocial behavior*, which includes social skills (i.e., peer-pressure resistance), self-esteem, attitudes towards drug use, attitudes towards police, and family bonding. The program is normally taught by a police officer; and the core curriculum has 17 lessons, usually offered once a week for 45 to 60 minutes [3]. This typically results in an expensive program. According to Dukes *et al.* [4], the average cost per uniformed police officer approached $50,000 per year, and the cost per student was at least $100. In recent years, the annual federal expenditures on the D.A.R.E. program reached $750 million [5]. Nonetheless, the parents were positive about the D.A.R.E. program because they viewed the D.A.R.E. officers as effective educators [6]; the classroom teachers’ also gave their high ratings to teacher-officer interaction, role-playing exercises, and graduation ceremony [7].

Although there seems to be great support for the D.A.R.E. program, the literature revealed inconsistent research results concerning the program’s effectiveness. For example, some studies found that the D.A.R.E. program did not work [5, 8–15]; whereas other researchers claimed that the D.A.R.E. program was effective [16–23]. More specifically, the literature over the past two decades showed that the D.A.R.E. program had short-term effects on some of the outcomes on drug use and psychosocial behavior [4, 6, 20–23], but the D.A.R.E. program has not shown long-term (i.e., more than one year) effects on drug use [10, 24–36].

Considering the tremendous investment of time and money in the D.A.R.E. program, these inconsistent findings necessitate a conclusive synthesis of the research to assess the effectiveness of the program. To date, only two published research syntheses or meta-analytic reviews exist that solely focused on evaluating the effectiveness of the D.A.R.E. program [5, 10]. Unfortunately, the two meta-analyses have some limitations. For example, Ennett *et al.* [10] examined only eight studies, and four of them were annual reports produced exclusively for the D.A.R.E. agencies, which was called into question [5, 37]. West and O’Neal [5], on the other hand, reviewed the effects of the D.A.R.E. program only on drug use. Additionally, neither of the two meta-analyses explored the relationships between the study characteristics and the outcome measures. Finally, the two reviews analyzed the outcomes either independently [10] or as one simple sum of drug use measures [5]. Because the two major outcomes, drug use and psychosocial behavior, are conceptually unique but realistically related to one another, a multivariate meta-analysis [38] serves as a more appropriate analytical approach to analyze the multiple outcomes simultaneously.

The purpose of this multivariate meta-analytic review was to: (a) quantitatively synthesize updated evaluation studies of the D.A.R.E. program, and (b) simultaneously synthesize all the outcomes of the D.A.R.E. program. Specifically, this review addressed the following three research questions: (a) Did the effects of the D.A.R.E. program on the outcomes vary across the studies? (b) What was the overall effect of the D.A.R.E. program on the outcomes? (c) What study characteristics explained the variation of the effects of the D.A.R.E. program on the outcomes?

## 2. Method

### 2.1. Literature Search

Using the terms “Drug Abuse Resistance Education,” “D.A.R.E.,” and “school-based drug prevention program” as keywords, an extensive literature search in the PsycINFO, MEDLINE, ERIC, PUBMED, SOCIAL SCIENCE INDEX, SOCIAL SCIENCE INDEX CITATION, and DIGITAL DISSERATIONS databases produced 198 relevant studies published between January 1983 and December 2005. Of the 198 studies, 73 were found to be quantitative studies on the D.A.R.E. program. After applying the criteria for inclusion described below, 20 final studies were selected for this meta-analysis.

### 2.2. Study Inclusion Criteria

The first criterion for inclusion required the study to have sufficient quantitative information for calculating the outcome measure or the effect size of the outcome: Cohen’s *d* [39]. Cohen’s *d* is a standardized mean difference between the treatment group and the control group. That is,

(1) $$d=\frac{{\bar{x}}_{t}-{\bar{x}}_{c}}{s_{p}},$$

where *x̄_t_* is the mean of the treatment group, *x̄_c_* is the mean of the control group, and *sp* is the pooled standard deviation. Whether the study utilized an experimental or quasi-experimental design was the second criterion because these designs are more rigorous and provide more valid research results than other less scientific designs. The third criterion necessitated that the study evaluated at least one of the outcomes on drug use and psychosocial behavior. The fourth and final criterion called for studies where the effect of the D.A.R.E. program could be independently evaluated. That is, whether the studies provided a D.A.R.E. treatment group and a comparable control group.

### 2.3. Recorded Variables

*Outcome measures.* The outcome measures for the present review were two sets of effect sizes: one was for drug use and the other for psychosocial behavior. Each effect size in the former set was the average Cohen’s *d* for all the available drug use outcomes (i.e., tobacco use, alcohol use, marijuana or other illicit drug use), and the latter for all the available psychosocial behavior outcomes (i.e., peer-pressure resistance, self-esteem, attitudes towards drug use, attitudes towards police, or family bounding). Different effect size measures calculated from various statistical methods in the studies were converted to Cohen’s *d*. In line with the previous reviews [5, 10], the Cohen’s *d*s of the outcomes across the studies were calculated at the *longest* follow-up, which ranged from 0 (i.e., right after the program) to 10 years.

*Study characteristics*. The following study characteristics were recorded for the analysis: Name of first author, year of publication, sample size, statistical method (e.g., descriptive statistics, general linear models, and multilevel models, which are in the order of methodological rigor), year of D.A.R.E. curriculum, follow-up time, proportion of female participants, and proportions of ethnic groups. The selection of the study characteristics was partially guided by the pervious reviews and partially based on common information available in the studies.

### 2.4. Coding Procedure

Each value of the variables of the study characteristics and outcome measures needed to be recorded or coded from the 20 studies. A concurrent double coding was performed independently by the researchers. Each researcher spent more than forty hours, equivalent to five full-time work days, on coding the 20 studies. Then, the researchers engaged in extensive discussions to compare every coded item. No variable was finalized until reaching an agreement.

### 2.5. Statistical Analysis

Descriptive analysis was first conducted for each of the two outcomes, drug use and psychosocial behavior, by calculating the unweighted mean effect size of the outcome. According to Cohen’s guideline [39], *d* = 0.20, 0.50, and 0.80 are considered small, medium, and large effect, respectively. 95% confidence intervals of the effect sizes were also computed. The confidence intervals showed whether the effect sizes were heterogeneous across the studies.

In terms of inferential analysis, a Hedges and Olkin’s [40] *Q*-statistic was computed for each of the two outcomes, drug use and psychosocial behavior. The test for the *Q*-statistic provided statistical evidence for the heterogeneity of the 20 studies. If the test was significant, a random-effects model was tested, and the weighted mean effect size was calculated to provide a more valid estimate for the mean effect size than the unweighted mean effect size from the descriptive analysis.

In the case of heterogeneous effect sizes, the study characteristics were entered into a *weighted regression model* to explain the variation in the heterogeneous effect sizes. Following Hedges’ [41] suggestion, the standard error used in the *t*-test for individual regression coefficient was adjusted as follows:

(2) $$\text{Adjusted}\;\;\;s.e.=\frac{s.e.}{\sqrt{MS_{\text{Error}}}},$$

where *s.e.* is the original standard error given by common computer programs, and *MS*_Error_ is the mean square value for errors from the analysis of variance for the regression given by the computer programs. Note that some study characteristics had missing values that resulted from unavailable information, and they were replaced by means [42] because the mean is the best single replacement value when no other information is available [43–45].

## 3. Results

### 3.1. Descriptive Analysis

Table 1 summarizes the 20 studies with the recorded variables. The unweighted mean effect sizes were 0.05 (ranging from –0.08 to 0.36) and 0.10 (ranging from –0.09 to 0.38) for drug use and psychosocial behavior, respectively. According to Cohen’s [39] interpretation, both the mean effect sizes were less than small although the mean effect size for psychosocial behavior was larger than that for drug use.

**Figure 1.**
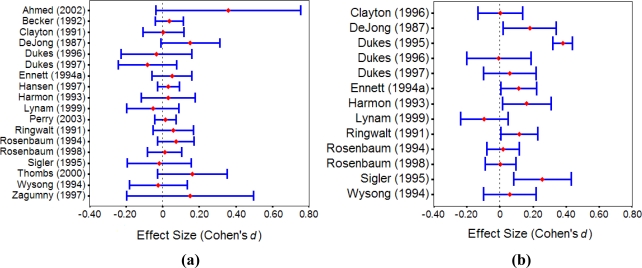
95% Confidence intervals of (a) drug use and (b) psychosocial behavior.

Figure 1 shows the 95% confidence intervals of the effect sizes for drug use and psychosocial behavior, respectively. From the confidence interval plots, we can see that the effect sizes across the studies were more heterogeneous for psychosocial behavior than those for drug use.

### 3.2. Inferential Analysis

*Test of homogeneity*. Under the null hypothesis of *H*_0_: θ_1_ = … = θ_20_ = θ, the Hedges and Olkin’s [40] *Q*-statistic values of *Q*_Total_ were 13.34 with *df* = 17 (*p* = 0.71) and 96.61 with *df* = 12 (*p* < 0.0001) for drug use and psychosocial behavior, respectively. The homogeneity test results showed that the effect sizes across the 20 studies were statistically heterogeneous for psychosocial behavior, but not for drug use. This inferential finding was consistent with the descriptive finding demonstrated in the confidence interval plots above. By testing a random-effects model for psychosocial behavior under *H*_0_: θ = 0, a *z* = 2.92 (*p* < 0.01) indicated that the weighted average effect size of the 20 studies from the random-effects model was statistically different from zero but was still 0.10, a less than small effect.

*Weighted regression analysis*. Because the effect sizes were heterogeneous for psychosocial behavior, a weighted regression analysis was conducted to identify the study characteristics that explained the heterogeneity. Table 2 displays the estimated coefficients of the significant characteristics of the studies from the weighted regression analysis with the adjusted standard errors (Eq. 2). From Table 2 we can see that five of the study characteristics significantly explained most of the variation of the effect sizes (*R*^2^ = 89.8%). Specifically, the longer follow-up time (*B* = –0.21, *t* = –2.49, *p* < 0.02) and the more rigorous statistical method (*B* = –0.13, *t* = –5.75, *p* < 0.001) the study used, the less effect of the D.A.R.E. program would be found for psychosocial behavior; whereas the later D.A.R.E. year (*B* = 0.04, *t* = 2.58, *p* < 0.02), the more White students (*B* = 0.01, *t* = 4.02, *p* < 0.002), and the more Black students (*B* = 0.01, *t* = 2.47, *p* < 0.03) the study had, the more effect of the D.A.R.E. program would have on psychosocial behavior.

## 4. Discussion

By including more updated studies and analyzing the study characteristics related to the outcomes of the D.A.R.E. program on both drug use and psychosocial behavior, this present multivariate meta-analysis provided a more comprehensive review than previous ones on the effectiveness of the D.A.R.E. program; and therefore, the present review helps us to better understand the widespread, expensive, but controversial D.A.R.E. program. The results of the present review revealed that the effects of the D.A.R.E. program on drug use were homogeneous but less than small, which confirmed the findings in the literature [5, 9–11, 13]. The present review also demonstrated that the effects of the D.A.R.E. program on psychosocial behavior were less than small but heterogeneous, which may explain why the D.A.R.E. program is still implemented in schools, welcomed by the parents, accepted by the communities, and supported by the government [6–7], despite some evidence that the D.A.R.E. program is not successful in reducing drug use among children.

For the heterogeneous effects of the D.A.R.E. program on psychosocial behavior, the present review found that the study characteristics explained most of the variation of the effects. The heterogeneous effects suggest that some studies showed larger effects than others. By examining the specific characteristics of the studies that had larger effects, which was executed in the weighted regression analysis, future program implementations can learn from those effective studies for improving the program effects. Among the significant study characteristics, follow-up time and statistical method were negatively related to the effects; and D.A.R.E. year, percent of White participants, and percent of Black participants positively related to the effects.

These findings provided some important implications. First, the validity of long-term effects might be threatened by maturity and history. This point was also noted in the previous reviews [5, 10] but without analyzing it. Second, more rigorous statistical methods that control for confounding variables could provide smaller, but more accurate estimates of the effect size. The similar methodological concerns about research design and sampling were also mentioned in Ennett *et al.* [10]. Third, specific, culturally-tailored D.A.R.E. programs might be needed to increase the effectiveness of the D.A.R.E. program on non-White and non-Black minorities. This implication is particularly meaningful for effectively implementing the program worldwide; and it would be interesting to explore the effectiveness of the D.A.R.E. program in other countries like Canada or Europe where the course is taught jointly with psychologists or specialists in different aspects of mental health and pedagogy. Fourth, the D.A.R.E. program has undergone several revisions since its inception [16]. The new D.A.R.E. program uses D.A.R.E. police officers as facilitators for student participation rather than as lecturers [16]. Some other significant revisions are to integrate high technology into the enhanced curriculum which includes internet safety, drive under influence, cyber bullying, and so on [2]. As such, it can be anticipated that the new revisions of the D.A.R.E. program would produce more effective outcomes. Therefore, it would be desirable to conduct a follow-up meta-analytic review on the effectiveness of the new D.A.R.E. programs. Last, it is worthy to note that the heterogeneity of the effects of the D.A.R.E. program on psychosocial behavior might come from other sources other than the study characteristics investigated in the present review. An example of such extra sources could be flaws in the implementation of the program.

In sum, the effects of the D.A.R.E. program appear to be different on drug use and psychosocial behavior. The results of the present review provide an evidence-based interpretation to the inconsistent conclusions found in the previous research that was conducted on the D.A.R.E. program. This study found that, on one hand, the D.A.R.E. program had a less than small effect on reducing drug use (Cohen’s *d* = 0.05); on the other hand, the school-based drug intervention program also had a less than small effect on improving psychosocial behavior (Cohen’s *d* = 0.10). The analysis from this review also identified areas in the new versions of the D.A.R.E. program that need improvement. It would be, however, more important if the new versions of the D.A.R.E. program could transform the improved psychosocial behavior into the students’ actions of reducing drug use—the ultimate outcome.

## Figures and Tables

**Table 1.** Study characteristics and effect sizes of the outcomes for the 20 studies.

Study	Study Characteristics									Effect Size (*d*)	
	D.A.R.E. (*n*)	Control (*n*)	Statistical Method	D.A.R.E. Year	Follow-up Time (Yr.)	F (%)	W (%)	B (%)	O (%)	Drug Use	Psycho-social Behavior
Ahmed (2002) [20]	208	28	G	1999	0	50.0	69.0	24.0	7.0	0.36	—
Becker (1992) [24]	1884	994	G	1989	0	—	—	—	—	0.04	—
Clayton (1991) [26]	1208	412	D	1987	2	51.0	75.0	22.0	2.0	0.01	—
Clayton (1996) [25]	858	285	M	1987	4	51.0	75.0	22.0	2.0	—	0.01
DeJong (1987) [21]	288	310	D	1985	1	—	—	—	—	0.15	0.18
Dukes (1995) [4]	2205	2181	D	1990	0	—	—	—	—	—	0.38
Dukes (1996) [28]	248	176	D	1987	3	—	—	—	—	–0.04	–0.01
Dukes (1997) [27]	356	264	D	1987	6	50.0	—	—	—	–0.08	0.06
Ennett (1994) [29]	715	608	G	1989	2	49.0	54.0	22.0	24.0	0.05	0.11
Hansen (1997) [30]	2393	1865	M	1990	3	57.8	71.1	27.6	1.4	0.03	—
Harmon (1993) [22]	341	367	D	1989	0	50.3	51.2	—	—	0.03	0.16
Lynam (1999) [32]	751	251	M	1987	10	57.0	75.1	20.4	4.5	–0.05	–0.09
Perry (2003) [13]	2518	2108	M	1999	1	48.4	67.3	7.5	25.2	0.01	—
Ringwalt (1991) [23]	685	585	G	1988	0	52.0	40.0	50.0	10.0	0.06	0.12
Rosenbaum (1994) [33]	859	725	G	1990	1	49.7	49.9	24.7	25.4	0.07	0.02
Rosenbaum (1998) [46]	975	823	M	1990	1	48.9	51.1	29.8	19.2	0.01	0.01
Sigler (1995) [47]	259	245	D	1990	0	—	—	—	—	–0.02	0.26
Thombs (2000) [34]	331	159	D	1992	8	58.6	90.4	5.5	4.1	0.16	—
Wysong (1994) [36]	288	331	D	1987	5	50.0	—	—	—	–0.02	0.06
Zagumny (1997) [48]	49	93	D	1991	5	48.0	—	—	—	0.15	—
*Mean*	*871*	*641*			*2.60*	*51.4*	*64.1*	*23.2*	*11.0*	*0.05*	*0.10*

*Note*. G = General linear model; D = Descriptive statistics; M = Multilevel models; F = Female; W = White; B = Black; O = Other race.

**Table 2.** Estimated coefficients from the weighted regression for psychosocial behavior (*R*^2^ = 0.898).

Study Characteristic	*B*	Adjusted *s.e.*	*t*	*p*
Follow-up Time (Yr)	–0.2082	0.0113	–2.4933	0.0283
Statistical Method	–0.1299	0.0226	–5.7527	0.0001
D.A.R.E. Year	0.0422	0.0164	2.5774	0.0242
% of White Students	0.0139	0.0035	4.0160	0.0017
% of Black Students	0.0090	0.0037	2.4724	0.0294

## References

[b1a-ijerph-06-00267] ^*^ References marked with an asterisk indicate studies included in the meta-analysis.

[1] Monteiro MG (2001). A World Health Organization perspective on alcohol and illicit drug use and health. Eur. Addict. Res.

[2] D.A.R.E. America (2007). D.A.R.E. America Annual Report.

[3] Bureau of Justice Assistance (1988). Implement Project D.A.R.E.: Drug Abuse Resistance Education.

[4.*] Dukes RL, Ullman JB, Stein JA (1995). An evaluation of D.A.R.E. (Drug Abuse Resistance Education) using a Solomon four group design with latent variables. Evaluation Rev.

[5] West SL, O’Neal KK (2004). Project D.A.R.E. outcome effectiveness revisited. Amer. J. Public Health.

[6] Donnermeyer JF (2000). Parents’ perceptions of a school-based prevention education program. J. Drug Educ.

[7] Donnermeyer JF, Wurschmidt TN (1997). Educators’ perceptions of the D.A.R.E. program. J. Drug Educ.

[8] Baum D (2003). Salt Lake City drops D.A.R.E. Rolling Stone.

[9] Burke MR (2002). School-based substance abuse prevention: Political finger-pointing does not work. Fed. Probat.

[10] Ennett ST, Tobler NS, Ringwalt CL, Flewelling RL (1994). How effective is drug abuse resistance education? A meta-analysis of Project D.A.R.E. outcome evaluations. Amer. J. Public Health.

[11] Glantz MD, Compton WM (2004). Mental health and substance abuse innovations: Issues of diffusion and adoption. Clin. Psychol.: Sci. Practice.

[12] Lindstrom P, Svensson R (1999). Evaluacion del programa preventivo D.A.R.E. en Suecia. [Evaluation of the Swedish school prevention program D.A.R.E.]. Adicciones.

[13.*] Perry CL, Komro KA, Veblen-Mortenson S, Bosma LM, Farbakhsh K, Munson KA, Stigler MH, Lytle LA (2003). A randomized controlled trial of the middle and junior high school D.A.R.E. and D.A.R.E. Plus programs. Arch. Pediatr. Adolesc. Med.

[14] Simkin DR (2002). Adolescent substance use disorders and comorbidity. Pediat. Clin. N. Amer.

[15] Szalavitz M (2002). D.A.R.E. to change. New Sci.

[16] Bowman DH (2002). Researchers see promising signs in D.A.R.E.’s new drug ed. program. Educ. Week.

[17] Eischens A, Komro KA, Perry CL, Bosma LM, Farbakhsh K (2004). The association of extracurricular activity participation with substance use among youth in the D.A.R.E. Plus Project. Amer. J. Health Educ.

[18] Miller KE (2003). Are D.A.R.E. and D.A.R.E. Plus effective prevention programs?. Amer. Fam. Physician.

[19] Ullman JB, Stein JA, Dukes R, Rose S, Chassin L, Presson CC, Sherman SJ (2000). Evaluation of D.A.R.E (Drug Abuse Resistance Education) with latent variables in context of a Solomon Four Group Design. Multivariate Applications in Substance Use Research: New Methods for New Questions.

[20.*] Ahmed NU, Ahmed NS, Bennett CR, Hinds JE (2002). Impact of a Drug Abuse Resistance Education (D.A.R.E) program in preventing the initiation of cigarette smoking in fifth- and sixth-grade students. J. Natl. Med. Assn.

[21.*] DeJong W (1987). A short-term evaluation of project D.A.R.E. (Drug Abuse Resistance Education): Preliminary indications of effectiveness. J. Drug Educ..

[22.*] Harmon MA (1993). Reducing the risk of drug involvement among early adolescents. Evaluation Rev.

[23.*] Ringwalt CR, Ennett ST, Holt KD (1991). An outcome evaluation of Project D.A.R.E. (Drug Abuse Resistance Education). Health Educ. Res.

[24.*] Becker HK, Agopian MW, Yeh S (1992). Impact evaluation of Drug Abuse Resistance Education (D.A.R.E.). J. Drug Educ..

[25.*] Clayton RR, Cattarello AM, Johnstone BM (1996). The effectiveness of Drug Abuse Resistance Education (Project D.A.R.E.): 5-year follow-up results. Prev. Med.

[26.*] Clayton RR, Cattarello AM, Walden KP (1991). Sensational seeking as a potential mediating variable for school-based prevention interventions: A two-year follow-up D.A.R.E. J. Health Commun.

[27.*] Dukes RL, Stein JA, Ullman JB (1997). Long-term impact of Drug Abuse Resistance Education (D.A.R.E.). Evaluation Rev.

[28.*] Dukes RL, Ullman JB, Stein JA (1996). A three-year follow-up of Drug Abuse Resistance Education (D.A.R.E.). Evaluation Rev.

[29.*] Ennett ST, Rosenbaum DP, Flewelling RL, Bieler GS, Ringwalt CL, Bailey SL (1994). Long-term evaluation of drug abuse resistance education. Addict. Behave.

[30.*] Hansen WB, McNeal RB (1997). How D.A.R.E. works: an examination of program effects on mediating variables. Health Educ. Behav.

[31] Kanof ME (2003). Youth Illicit Drug Use Prevention: D.A.R.E. Long-Term Evaluations and Federal Efforts to Identify Effective Programs.

[32.*] Lynam DR, Milich R, Zimmerman R, Novak SP, Logan TK, Martin C, Leukefeld C, Clayton R (1999). Project D.A.R.E.: No effects at 10-year follow-up. J. Consult. Clin. Psychol.

[33.*] Rosenbaum DP, Flewelling RP, Bailey SL, Ringwalt CL, Wilkinson DL (1994). Cops in the classroom: A longitudinal evaluation of Drug Abuse Resistance Education (D.A.R.E.). J. Res. Crime Delinq.

[34.*] Thombs DL (2000). A retrospective study of D.A.R.E.: Substantive effects not detected in undergraduates. J. Alcohol Drug Educ.

[35] Vastag B (2003). GAO: D.A.R.E. does not work. JAMA-J. Am. Med. Assn..

[36.*] Wysong E, Aniskiewicz R, Wright D (1994). Truth and D.A.R.E.: Tracking drug education to graduation and symbolic politics. Soc. Probl.

[37] Gorman DM (1995). The effectiveness of D.A.R.E. and other drug use prevention programs. Amer. J. Public Health.

[38] Becker BJ, Tinsley HEA, Brown SD (2000). Multivariate meta-analysis. Handbook of Applied Multivariate Statistics and Mathematical Modeling.

[39] Cohen J (1988). Statistical Power Analysis for the Behavioral Sciences.

[40] Hedges LV, Olkin I (1985). Statistical Methods for Meta-Analysis.

[41] Hedges LV, Cooper H, Hedges LV (1994). Fixed effects models. The Handbook of Research Synthesis.

[42] Pigott TD, Cooper H, Hedges LV (1994). Methods for handling missing data in research synthesis. The Handbook of Research Synthesis.

[43] Hair JF, Anderson RE, Tatham RL, Black WC (1998). Multivariate Data Analysis.

[44] Mertler CA, Vannatta RA (2005). Advanced and Multivariate Statistical Methods: Practical Application and Interpretation.

[45] Tabachnick BG, Fidell LS (2007). Using Multivariate Statistics.

[46.*] Rosenbaum DP, Hanson GS (1998). Assessing the effects of school-based drug education: A six-year multilevel analysis of Project D.A.R.E. J. Res. Crime. Delinq.

[47.*] Sigler RT, Talley GB (1995). Drug abuse resistance education program effectiveness. Amer. J. Police.

[48.*] Zagumny MJ, Thompson MK (1997). Does D.A.R.E. work? An evaluation in rural Tennessee. J. Alcohol Drug Educ.

